# The Making of a President: An Interview with Shirley Tilghman

**DOI:** 10.1371/journal.pgen.0020082

**Published:** 2006-06-30

**Authors:** Jane Gitschier

To see one of our own at the helm of a major university is unquestionably inspirational. And for those of us with two X chromosomes, perhaps even more so. I tried to imagine myself in the role of laboratory Cinderella: plucked from the work-a-day world of robocyclers and biohazard waste to a realm of literati, artists, and trustees, gliding into, if not glass slippers, pantyhose and pumps and a far spiffier wardrobe, my voice transformed from the constrained format of a journal article to one that can carry sway in every aspect of our culture.

This transition must have been second nature to Shirley Tilghman, President of Princeton University (see Video S1). She is the model of grace, intellect, perseverance, womanhood, and tact. Just 24 hours before my interview with her was scheduled, Lawrence Summers (President of Harvard) had resigned, and I half anticipated that I might be bumped for a sound bite. Indeed, her phone was ringing off the hook, but our interview held fast.

I had driven to New Jersey from my father's house in Pennsylvania the night before. As I turned north on Highway 206, I took in the Lawrenceville playing fields on the right and the stately old homes on the left, recalling a day 36 years ago when I followed the Orange Key Tour, the last time I had visited Princeton. I recognized Nassau Hall, the pair of bronze tigers protecting a set of massive, deep-blue doors embedded in ivy-covered stone walls. I approached the old building, walked across the marble foyer to an unmarked door, and fell into a cozy warren, made even more inviting by soft green colors and a bowl of chocolates.

Shirley met me and led me down a little set of steps to her office. Journal covers prominently displayed near her desk remind her of her former life, as does a bronzed Rainin pipetman with a plaque that reads “I'm a genius!”, given to her by her lab when she became president. I told her the topics I wanted to cover in our interview.

**Jane Gitschier:** …and third, I want to talk about Princeton!

**Shirley Tilghman:** Yeah!

**JG:** So why don't we start with that? I have read a couple of interviews with you, and I was so impressed by your comments about how you just love the institution. You've been here for almost 20 years and president for almost five. Tell me, why do you love it here?

**ST:** There are really two answers. First, it's a place that is always striving for excellence, and excellence with integrity. I've loved that since the day I arrived.

The second is that it is a place that is run by the faculty. Here I am, as testimony to that! In fact, if you look at the history of the university, there has been only one president in recent history that did not come from the faculty. It is a place that respects scholarship, respects intellectuals, and believes that a university is best run by people who grew up caring about the life of the mind.

**JG:** That's wonderful!

**ST:** Yes, and the third thing I love about Princeton is that the students are just spectacular. They challenge you and make you better. Interacting with them in my old life in the lab and in my new life in Nassau Hall, every day is just fun.

**JG:** How many students are there?

**ST:** There are now about 4,700 undergraduates and we're going up to 5,100. We're in a process of expansion, as a matter of fact.

**JG:** Why do you feel the need to increase the undergraduate population?

**ST:** There were a number of reasons, but probably the most important was that during the last 30 years, we had kept the population of the undergraduate student body relatively constant, while we were growing the faculty at a rate of about 1% per year. And we had reached a point where the student-faculty ratio was under six, which is extraordinary for a university, particularly a research university.

Second, we have the largest endowment per student in the country. I think that is a joy, to a president, but it also creates a responsibility. I believe that the trustees, who ultimately made the decision to expand, believed that if we have the capacity in both faculty and resources to educate more students, we should.

**JG:** And the graduate student body?

**ST:** We have about 2,100 graduate students. The graduate school student population has always been roughly a third the size of the undergraduate student body. We can do that because, of course, we have very few professional schools.

**JG:** Do you think that there would be some virtue to having a law school or a medical school affiliated with the university, or is that just not brought up?

**ST:** It comes up with a periodicity of about 25 years.

**JG:** Pretty slow!

**ST:** But it is studied periodically. The trustees cogitate about it, and, at the end, they decide that one of our distinctive characters is that we are, as an institution, so focused on two things: (1) the best quality undergraduate education that we can deliver, and (2) really stellar PhD production. The fact that we're so focused is one of our strengths.

**JG:** I read one account of how you had been on the search committee [for the president] and then, while you went off to teach a class, the other members decided to offer you the job!

**ST:** It was more, “Shouldn't we think about the possibility that she might be a candidate?” They were still six weeks away from making their final decision, but that is roughly how it happened.

**JG:** I'm thinking of how weird this is, to go from being a professor with almost no administrative experience….

**ST:** What were they thinking?!!

**JG:** What do you think they saw in you? Did you have a vision that you articulated to them?

**ST:** The search committee met a lot for the first four months. We spent a lot of time talking about the presidency and about Princeton. We traveled together, in pairs, all over the country interviewing other presidents, leaders in the scientific community, and those who would have perspectives on what a university is going to face in science.

So this group really knew each other well by the time they asked me to step down [from the committee]; we had all been very frank with one another. This process was highly confidential, so people felt free to express their views. I suspect that by the time they asked me to step off the committee, they had a pretty good idea of how I thought about Princeton, what the challenges were likely to be, what I thought we should be looking for in a candidate. I had supported some of the candidates who were clearly rising to the short list, so I think they also had a sense of my taste.

**JG:** At the time, what did you think were the challenges, opportunities, and future directions for Princeton?

**ST:** Certainly, at the time I felt strongly that it would be helpful to have someone who could grapple with some of the challenges that research universities face—to sustain what I think is our critical role as innovation engines for the country. And as we look at what has happened in Washington over the last four years, I think this concern has been borne out—despite what we heard in the State of the Union address—“Show me the money!” I was worried that as science became more expensive and more complicated, how could we continue to provide support in universities? After all, there is nothing sacrosanct about the idea that fundamental research must occur in universities. It could have easily been decided in the late '40s that federally funded research should occur in research institutes or in national labs. I think, though, the connection between science and teaching has been so productive for this country.

The other thing that I was concerned about on the search committee was how a place like Princeton, which has both the joy and the burden of great tradition, balance respect for that tradition with the need to move forward.

**JG:** So, how do you like your job?

**ST:** I love my job!

**JG:** What do you love about it?

**ST:** Well, it goes back to your very first question. I love this place. You can effect change in real time. You can see the effect of what you're doing, from recruiting a senior faculty member down to simple things like hearing about some ridiculous rule and changing it to make everybody's life easier.

**JG:** Power has its merits.

**ST:** You have to be careful, of course, because you don't want to become a rogue elephant.

Another reason that this is such a great job, which I didn't fully anticipate, is the opportunities it has given me to broaden my intellectual horizons. For example, I was in New York City yesterday talking to the Commissioner of Cultural Affairs about how our new arts initiative can engage New York City more effectively. This is an area of the university world that I knew very little about until I became president.

**JG:** Do you ever feel overwhelmed by all the different tasks you need to attend to, or is it manageable?

**ST:** It's manageable, because Princeton is so focused on trying to be very good at a small number of things.

**JG:** You are still in the molecular biology department?

**ST:** As far as I know! They haven't disowned me yet.

**JG:** You don't teach any more, do you?

**ST:** I do teach! I teach part of the introductory molecular biology class to freshman and sophomore students. And I advise senior theses and junior independent work.

**JG:** So, you still have a laboratory!

**ST:** I just sent off my last paper to *Genes and Development,* which I hope will publish it. My last post-doc is about to leave, and then I'll close the lab down.

As you can imagine, it is not possible to run a lab and run this university at the same time. That was one of the clear implications of agreeing to do this [job].

**JG:** When I've told other women I am about to interview you, they say something to the effect of “Oh, she's my role model—have you heard ‘The Speech'?” I've never had the benefit of hearing you speak about women in science. Perhaps you could condense it into a few minutes of tape! Or perhaps I can ask a few questions and maybe the speech will come out.

**ST:** Let's try that!

**JG:** OK, let's start then with your early career. You were an assistant professor at Temple [University] and then at Fox Chase [Cancer Center] in Philadelphia. Were you then looking at Princeton because of the proximity?

**ST:** No, I began to think about leaving Fox Chase when David Baltimore tried to recruit me to the Whitehead [Institute], and I looked at it very seriously. I saw a great deal that I found enormously attractive, including the chance to be with Rudi Jaenisch, who is in my field and whom I adore. It was after it became known that I was looking that Arnie Levine called and asked if I would also look at Princeton.

At the end of the day, it was really a family decision to come to Princeton. I was single by this point, with two little kids. As I tried to figure out how I could afford to live in Boston, send the children to private school, commute to the Massachusetts Institute of Technology (MIT), it just didn't compute. Princeton made everything possible—a little town with really good public schools, everything was within three minutes: three minutes to home, three minutes to the pediatrician, three minutes to the primary school, the nursery school. And it was a great university.

**JG:** How did you manage? Your children were little.

**ST:** They were age four and six years.

**JG:** Did you have someone live in your home?

**ST:** No, because it wasn't big enough. Over the years, I had a whole series of solutions to the problem you are identifying. If I had to do it again, I would definitely hire a live-in person. When I look back on the past, I think it was a terrible mistake not to do it.

**JG:** I'm sympathetic to your history because I am also a single parent. We make these decisions so that we can function.

**ST:** It's really true. If I had been making that decision purely on the quality and critical mass of science, I would have gone to MIT. You do what you have to do. And I've never regretted it for one minute. Princeton was exactly the right place for me at that time. And this has proven to be the right place for me in the long run, too!

**JG:** Have we started to touch on “The Speech”? Are we getting close??

**ST:** One version of “The Speech” is on the Princeton President's Web site (http://www.princeton.edu/president/speeches). The one that comes closest to what people are talking about is the one I gave at Columbia [University] last year about this time [March 24, 2005].

**JG:** What was the topic?

**ST:** It was about the future prospects for women in science—why it's important that women are represented in science, why it hasn't happened until now, and what we need to do to change things.

**JG:** One thing that I've found incredibly helpful has been to have Howard Hughes Medical Institute (HHMI) funding.

**ST:** Yes, HHMI was tremendously good to me. I wouldn't be studying genomic imprinting if it hadn't been for its support.

**JG:** And why is that?

**ST:** I was studying this *H19* gene, and everyone thought I was completely crazy. If I had had to write an National Institutes of Health (NIH) grant to defend it in 1985, I probably would have had a really hard time. The HHMI money, as you well know, allows you to work on whatever you want to work on.

**JG:** Since you brought it up, how did you get interested in *H19*?

**ST:** Because it was a mystery!

**JG:** It was so different from what you had been doing previously, working on alpha-fetoprotein (AFP).

**ST:** Yes, but there is a connection. My first real foray into pure mouse genetics was to study two trans-acting loci that regulated the levels of postnatal levels of AFP during mouse development. One recessive mutation caused a significantly elevated level of AFP mRNA after birth compared to wild-type strains. The question we asked was whether this trans-acting locus, called raf, regulates genes other than AFP. So we did a 1980s version of microarray analysis. We looked for genes whose hepatic expression declined after birth in a strain-specific manner, in a manner parallel to AFP. *H19* came out of that screen.

Remember those little Millipore filters that had grids on them and you'd grow cDNA-containing bacterial colonies on them and then hybridize them? Grunstein-Hogness! So that's what we did. *H19* was the H row, 19th spot. We had made a liver cDNA library and we were screening through them.

*H19* was incredibly abundant and we showed that it was developmentally regulated, just like AFP. So this wonderful student, Vassilis Pachnis, who's gone on to be very successful at Mill Hill [MRC National Institute of Medical Research] in London, sequenced it, before machines. He kept hitting stop codons, and I kept saying, “Vassilus, you're making sequencing mistakes. It must have an open reading frame. There is no precedent for this for a spliced polyadenylated RNA.” So this poor guy went back and continued to sequence and finally persuaded me that this was a highly abundant, spliced, capped RNA with no reading frame. And that was the mystery!

I kept plugging away on it, thinking there must be something here. Given its abundance and its tight regulation, this can't be a garbage RNA.

**JG:** Well, I can see why you thought the NIH wouldn't fund it, because we are so entrenched in dogma.

**ST:** Right. That is might be imprinted was just a lucky guess in the early '90s. It was one of these funny moments in science where you just have a leap of faith. You connect things and have a “eureka” moment. When I guessed it might be imprinted, I asked Marisa Bartolomei to check it out. It was the third gene shown to be imprinted, after insulin-like growth factor 2 and its binding protein, Igf2r.

**JG:** Those genes are adjacent.

**ST:** That's what got me going. At the end of the day we knocked out *H19* and there was no phenotype. If I have one regret about my career, it is that I had to stop before I figured out what *H19* does!

Unfortunately this little filter array name [*H19*] has stuck on it.

**JG:** At least it's not an acronym that stands for something you can never remember, like so many other mammalian genes.

**ST:** Exactly. I always think the fly guys had it right. Give genes very distinctive names.

**JG:** I agree, but as a human geneticist, when you have to tell parents, “We're sorry but your child has a mutation in sonic hedgehog,” there's something that seems weird about that to me. It sounds so trivial for something that has such serious import. But the spirit of the Drosophila people is fabulous!

But let's go back to your post-doc, working with Phil Leder.

**ST:** It was a wonderful time. The NIH was the biggest sandbox I had ever played in. At that time, it was so exciting. There was literally no area of life sciences that you could become interested in and not find somebody at the NIH who was knowledgeable. So it was exciting and intellectually engaging.

And Phil was heaven to work for. He was very supportive and yet gave me independence. Cloning the globin gene, as you can imagine, was so exciting and then discovering that its structure was so different [having intervening sequences] from what everyone had anticipated was thrilling. It was one of those “hairs on the back of your neck” moments when you know you've discovered something important.

Phil has been a model mentor to me ever since. There is no turn in my career at which I haven't consulted him—including whether to take this job—and when he hasn't offered assistance, advice, moral support.

**JG:** You are lucky to have that. Very few of us do. I think we need something to change so that women don't say “I can't do this” even before they give it a shot. Have you been able to affect the work culture at all?

**ST:** I've increasingly started to worry that we spend too much time talking about how difficult science is and not enough talking about the fact that despite the difficulty—and I would never say it is not difficult because it is difficult—women can succeed and thrive. They can balance successful science careers and children. Think of the number of women that we know in that category.

Too often, I think, our graduate students and post-docs only hear the sob stories. They hear that it's so hard and impossible. I think if you hear that long enough, you inevitably become discouraged and conclude that you can't be so special that you'll be able to overcome all these barriers.

**JG:** Who do you think they are hearing this from?

**ST:** I think they are hearing this from us!

**JG:** Aha—this is where “The Speech” comes in!

**ST:** Yes. In the Columbia speech, I began with a whole series of pictures of extremely successful women in science who have had children and are now luminaries. And I ended with young women in science, just post post-doc, heading off to what I think are going to be the same kind of careers. I tried to make the point that it's not that it isn't hard. It is hard! It's hard for women to be doctors and lawyers. The more I learn about it, the more I think there is nothing unique about science that makes it more difficult for women to have a career than any other field.

It is possible. What we should be talking about more are the things you and I were just talking about. What do you need to have in your life to make it possible to have a career and a fulfilling life outside the lab? How do you organize childcare? How do you take maternity leave and keep the lab moving along?

Here at Princeton, we just created a backup [child] care benefit for all employees and students. You can call this company at a moment's notice and say, “It's a snow day tomorrow, I've got to have someone at my house at 8 o'clock in the morning.”

**JG:** Oh, that's great!

**ST:** Isn't that great? And it costs US$4 an hour. We are subsidizing it. But imagine the pressure it takes off people!

**JG:** You must have very little time to relax now and your evenings must be booked!

**ST:** Yes, that's one thing that has been very different. It's the nights, the dinners, the receptions. On the other hand, I often end up going to a student play or a dance recital or a basketball, lacrosse, or soccer game, and that is fun! These students come here and they entertain me! This is true! I don't find that it is stressful at all.

**JG:** Of course, your children are grown up now. And that is hugely different.

**ST:** Hugely different! I wouldn't have taken this job ten years ago. Couldn't have done it. Part of it is managing your limits. I had a little sign above my phone in the Lewis Thomas Lab that said “Just say no!” It's the only thing I've agreed with Nancy Reagan about.

**JG:** Well, I'm awfully glad you didn't say “No” to me! 

## Supporting Information

Video S1 Interview with Shirley Tilghman (8.6 MB MOV)

**Figure:**
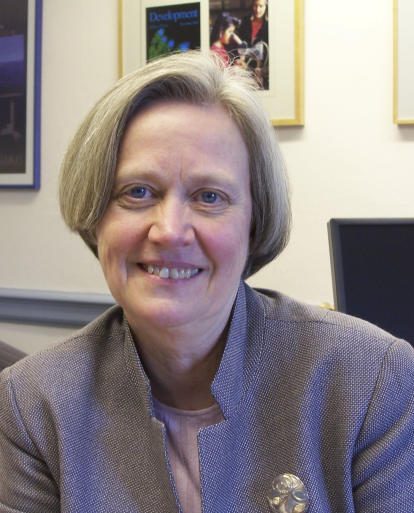
Shirley Tilghman

## Abbreviations

AFP: alpha-fetoprotein
NIH: National Institutes of Health

